# The National Patient-Centered Clinical Research Network (PCORnet) Bariatric Study Cohort: Rationale, Methods, and Baseline Characteristics

**DOI:** 10.2196/resprot.8323

**Published:** 2017-12-05

**Authors:** Sengwee Toh, Laura J Rasmussen-Torvik, Emily E Harmata, Roy Pardee, Rosalinde Saizan, Elisha Malanga, Jessica L Sturtevant, Casie E Horgan, Jane Anau, Cheri D Janning, Robert D Wellman, R Yates Coley, Andrea J Cook, Anita P Courcoulas, Karen J Coleman, Neely A Williams, Kathleen M McTigue, David Arterburn, James McClay, PCORnet Bariatric Surgery Collaborative

**Affiliations:** ^1^ Department of Population Medicine Harvard Medical School and Harvard Pilgrim Health Care Institute Boston, MA United States; ^2^ Northwestern Medicine Chicago, IL United States; ^3^ Vanderbilt University Nashville, TN United States; ^4^ Kaiser Permanente Washington Health Research Institute Seattle, WA United States; ^5^ Ochsner Surgical Weight Loss Center New Orleans, LA United States; ^6^ COPD Foundation Washington, DC United States; ^7^ Duke Clinical & Translational Science Institute Durham, NC United States; ^8^ Department of Surgery University of Pittsburgh Medical Center Pittsburgh, PA United States; ^9^ Department of Research and Evaluation Kaiser Permanente Southern California Pasadena, CA United States; ^10^ Community Partners' Network Nashville, TN United States; ^11^ Department of Medicine University of Pittsburgh Pittsburgh, PA United States; ^12^ University of Nebraska Medical Center Omaha, NE United States; ^13^

**Keywords:** obesity, bariatric surgery, comparative effectiveness, real-world evidence, weight loss

## Abstract

**Background:**

Although bariatric procedures are commonly performed in clinical practice, long-term data on the comparative effectiveness and safety of different procedures on sustained weight loss, comorbidities, and adverse effects are limited, especially in important patient subgroups (eg, individuals with diabetes, older patients, adolescents, and minority patients).

**Objective:**

The objective of this study was to create a population-based cohort of patients who underwent 3 commonly performed bariatric procedures—adjustable gastric band (AGB), Roux-en-Y gastric bypass (RYGB), and sleeve gastrectomy (SG)—to examine the long-term comparative effectiveness and safety of these procedures in both adults and adolescents.

**Methods:**

We identified adults (20 to 79 years old) and adolescents (12 to 19 years old) who underwent a primary (first observed) AGB, RYGB, or SG procedure between January 1, 2005 and September 30, 2015 from 42 health systems participating in the Clinical Data Research Networks within the National Patient-Centered Clinical Research Network (PCORnet). We extracted information on patient demographics, encounters with healthcare providers, diagnoses recorded and procedures performed during these encounters, vital signs, and laboratory test results from patients’ electronic health records (EHRs). The outcomes of interest included weight change, incidence of major surgery-related adverse events, and diabetes remission and relapse, collected for up to 10 years after the initial bariatric procedure.

**Results:**

A total of 65,093 adults and 777 adolescents met the eligibility criteria of the study. The adult subcohort had a mean age of 45 years and was predominantly female (79.30%, 51,619/65,093). Among adult patients with non-missing race or ethnicity information, 72.08% (41,248/57,227) were White, 21.13% (12,094/57,227) were Black, and 20.58% (13,094/63,637) were Hispanic. The average highest body mass index (BMI) recorded in the year prior to surgery was 49 kg/m^2^. RYGB was the most common bariatric procedure among adults (49.48%, 32,208/65,093), followed by SG (45.62%, 29,693/65,093) and AGB (4.90%, 3192/65,093). The mean age of the adolescent subcohort was 17 years and 77.5% (602/777) were female. Among adolescent patients with known race or ethnicity information, 67.3% (473/703) were White, 22.6% (159/703) were Black, and 18.0% (124/689) were Hispanic. The average highest recorded BMI in the year preceding surgery was 53 kg/m^2^. The majority of the adolescent patients received SG (60.4%, 469/777), followed by RYGB (30.8%, 239/777) and AGB (8.9%, 69/777). A BMI measurement (proxy for follow-up) was available in 84.31% (44,978/53,351), 68.09% (20,783/30,521), and 68.56% (7159/10,442) of the eligible adult patients at 1, 3, and 5 years of follow-up, respectively. The corresponding proportion was 82.0% (524/639), 49.9% (174/349), and 38.8% (47/121) in the adolescent subcohort.

**Conclusions:**

Our study cohort is one of the largest cohorts of patients with bariatric procedures in the United States. Patients are geographically and demographically diverse, which improves the generalizability of the research findings and allows examination of treatment effect heterogeneity. Ongoing and planned investigations will provide real-world evidence on the long-term benefits and risks of these most commonly used bariatric procedures in current clinical practice.

## Introduction

As severe obesity has increased in prevalence, the use of bariatric surgery has expanded considerably over the past 20 years. Because of this expansion and the rapid shifts in the types of bariatric procedures performed in recent years—from predominantly Roux-en-Y gastric bypass (RYGB) in the early 2000’s, shifting towards greater use of adjustable gastric banding (AGB) by early 2010’s, and then to predominantly sleeve gastrectomy (SG) currently [1-3]—long-term data comparing the effectiveness and safety of different procedures on sustained weight loss, comorbidities, and adverse effects are limited. In addition, prior studies have included insufficient numbers of patients to examine differential outcomes within important patient subgroups. More data are needed in larger, more broadly representative samples with long-term follow-up to help inform clinical decisions about bariatric procedure selection in various patient sub-populations (eg, individuals with diabetes, older patients, adolescents, and minority patients).

In 2014, the Patient-Centered Outcomes Research Institute (PCORI) launched the National Patient-Centered Clinical Research Network (PCORnet) to support studies that address questions important to patients [4]. PCORnet is a distributed data network that includes 13 Clinical Data Research Networks (CDRNs) and 20 Patient-Powered Research Networks, making it one of the largest research consortia in the United States. It currently includes electronic health record (EHR) or administrative claims data from more than 100 million individuals and has access to over 40 million patients who could be recruited into pragmatic clinical trials. PCORnet data is stored at individual participating sites in a common data format [5].

Initiated in 2016, the PCORnet Bariatric Study (PBS) is one of the first 2 multi-CDRN observational studies conducted within the network [6]. A group of patients, clinicians, and researchers developed the study aims [7]. The cohort was set up with 2 major goals. The first was to evaluate the comparative effectiveness and safety of AGB, RYGB, and SG, the 3 most commonly performed bariatric procedures in contemporary clinical practice. The second goal was to demonstrate PCORnet’s potential as a national resource for evidence generation. Here, we describe the design and early descriptive results of the study.

## Methods

### Data Sources

A total of 42 health systems from 11 CDRNs participated in this descriptive study (Textbox 1). Of the 2 non-participating CDRNs, 1 outpatient-focused network deferred due to insufficient number of bariatric patients and the other network was not yet founded at the time that the PBS was proposed. The participating health systems are geographically diverse and provide care to demographically heterogeneous populations.

As part of its efforts to facilitate rapid and efficient studies drawing from multiple data sources, PCORnet standardized the EHR data from the participating health systems by implementing a common data model (CDM). Table 1 describes the specific data domains extracted from the EHRs. These domains include patient demographics, encounters with healthcare providers, diagnoses recorded and procedures performed during these encounters, vital signs, laboratory test results, and mortality (obtained from other sources in some CDRNs).

**Eleven participating PCORnet Clinical Data Research Networks and data-contributing sites in the PCORnet Bariatric Study. Johns Hopkins University and Health System, UPMC Health Plan, and Boston HealthNet did not contribute data for this paper but will for future analyses.**

Clinical Data Research Network (CDRN) and the corresponding data-contributing sites

- Chicago Area Patient-Centered Outcomes Research Network (CAPriCORN)
  - Loyola Medicine
  - Northwestern Medicine
  - University of Chicago Medical Center
  - University of Illinois Hospital & Health Science System
- Greater Plains Collaborative (GPC)
  - Marshfield Clinic
  - University of Texas Southwestern Medical Center
  - University of Iowa Healthcare
  - University of Kansas Medical Center
  - University of Wisconsin - Madison
  - University of Nebraska Medical Center
- Kaiser Permanente & Strategic Partners Patient Outcomes Research To Advance Learning (PORTAL)
  - Kaiser Permanente Washington Health Research Institute (formerly Group Health Research Institute)
  - HealthPartners Research Foundation
  - Kaiser Permanente Colorado
  - Kaiser Permanente Mid-Atlantic
  - Kaiser Permanente Northwest
  - Kaiser Permanente Southern California
- Mid-South
  - Greenway
  - University of North Carolina
  - Vanderbilt University Medical Center
- New York City Clinical Data Research Network (NYC-CDRN)
  - Mount Sinai
  - New York University
  - Weill Cornell
  - Montefiore/Einstein
- OneFlorida Clinical Research Consortium
  - University of Florida Health
  - Orlando Health
  - Tallahassee Memorial Health System
- PaTH Towards a Learning Health System Clinical Data Research Network (PaTH)
  - Geisinger Health System
  - Johns Hopkins University and Health System
  - Penn State College of Medicine, Penn State Milton S. Hershey Medical Center
  - Temple Health System, Lewis Katz School of Medicine at Temple University
  - University of Pittsburgh and University of Pittsburg Medical Center (UPMC)
  - UPMC Health Plan
  - University of Utah and University of Utah Health Care
- A Pediatric Learning Health System (PEDSnet)
  - Cincinnati Children’s Hospital Medical Center
  - Nemours
  - Nationwide Children’s Hospital
- Patient-Centered SCAlable National Network for Effectiveness Research (pSCANNER)
  - University of California Irvine
  - University of California Los Angeles
- Research Action for Health Network (REACHnet)
  - Baylor Scott & White Health
  - Ochsner Health System
  - Tulane University
- Scalable Collaborative Infrastructure for a Learning Healthcare System (SCILHS)
  - Beth Israel Deaconess Medical Center
  - Boston HealthNet
  - Partners Health
  - Wake Forest Baptist Hospital

**Table 1:** Data elements collected by the PCORnet Bariatric Study from the PCORnet common data model.

Domain	Description	Applicability for PCORnet Bariatric Study
Demographic	Contains 1 record per patient with key demographic variables.	Age at surgery, sex, race, and Hispanic ethnicity are captured.
Encounter	Contains 1 record for each time a patient sees a provider in ambulatory setting or is hospitalized; multiple encounters per day are possible if they occur with different providers or in different care settings.	Encounter type are used to identify initial bariatric procedures and all subsequent complications and procedures during the follow-up period. We have captured data from all encounter types including inpatient, outpatient, and emergency room visits.
Diagnosis	Contains all uniquely recorded diagnoses for all encounters. Each diagnosis is associated with a specific patient and encounter.	Diagnosis codes and associated encounter dates are used to establish medical history prior to surgery^a^.
Procedure	Contains all uniquely recorded procedures for all encounters. Each procedure is associated with a specific patient and encounter.	Procedure codes and associated encounter dates are used to establish bariatric surgery dates and any re-operations, revisions, or operative complications.
Vitals	Contains 1 record per height or weight result. Multiple measurements per encounter are recorded as separate measures.	Height and weight are captured for body mass index; blood pressure and tobacco use information is also available.
Lab Results	Contains 1 record per laboratory result.	The common data model currently contains a limited number of laboratory tests; glycated hemoglobin (HbA1c) is being collected and is required to identify diabetes outcomes in an ongoing analysis.
Death	Contains 1 record per patient for those who died.	Some health systems have existing linkages to state and national death indices; others will be funded to conduct linkages.

^a^We focused on extracting data on obesity-associated comorbidities and health conditions used to calculate the Charlson-Elixhauser combined comorbidity score.

**Figure 1:**
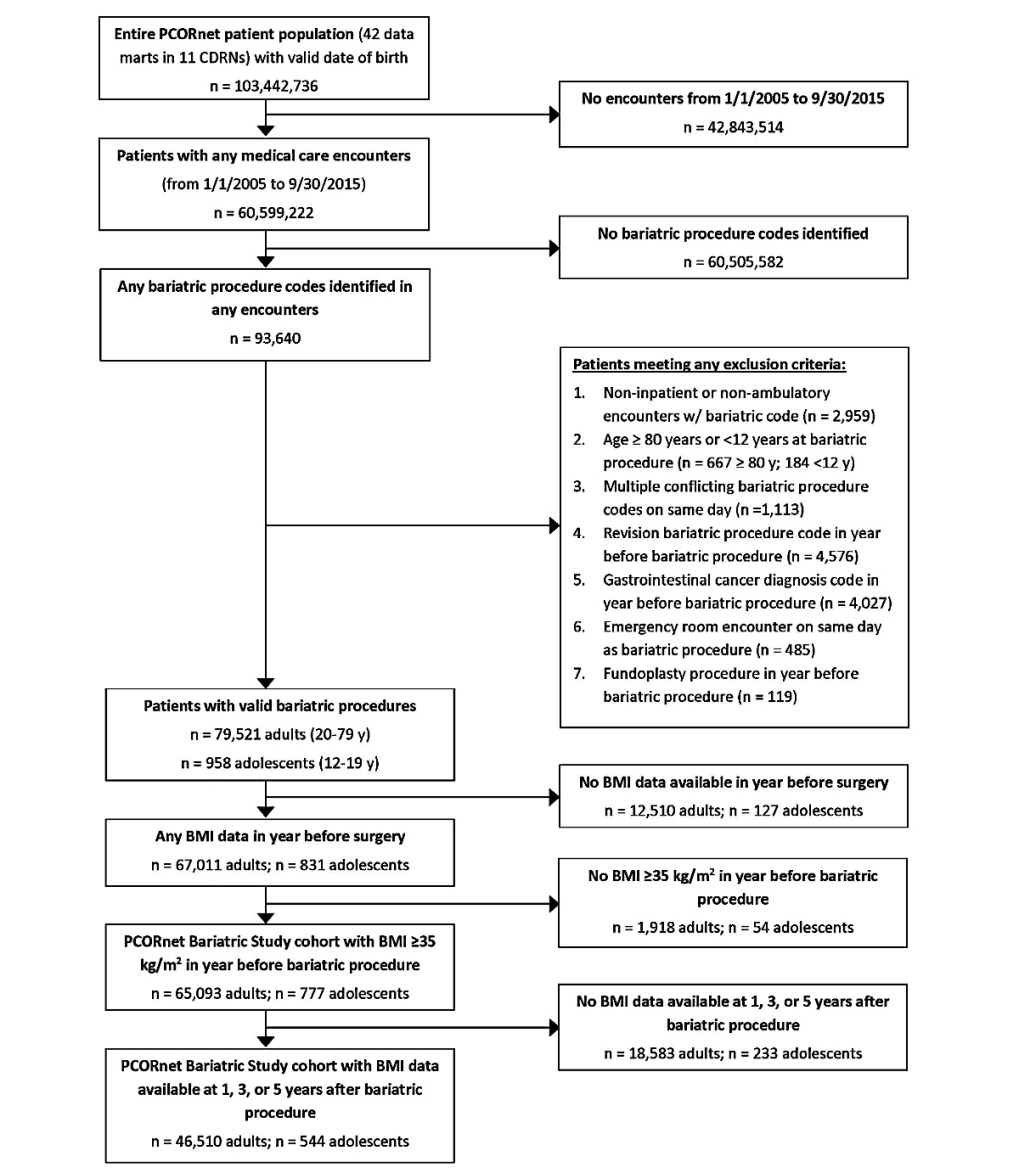
Flow diagram for identification of the PCORnet Bariatric Study cohort in 11 Clinical Data Research Network (CDRNs). BMI: body mass index.

### Cohort Identification

We identified adults (20 to 79 years old) and adolescents (12 to 19 years old) who underwent a primary (first observed) AGB, RYGB, or SG procedure between January 1, 2005 and September 30, 2015 in any of the 42 participating health systems. To be eligible for cohort inclusion, patients must have (1) at least 1 body mass index (BMI) measurement of 35 kg/m^2^ or more recorded in their EHRs in the year prior to the surgery (ie, baseline); (2) no prior revision bariatric procedure code during baseline; (3) no recorded gastrointestinal cancer diagnosis or fundoplasty procedure during baseline; (4) no multiple conflicting bariatric procedure codes on the same day; and (5) no emergency room encounter on the day of the index procedure (Figure 1). We excluded patients with missing baseline BMI, insufficient height or weight data to calculate baseline BMI, or baseline BMI less than 35 kg/m^2^ because guidelines recommend consideration of bariatric surgery for adult patients with severe obesity (BMI 40 kg/m^2^ or greater) or BMI 35 kg/m^2^ or greater plus comorbidity [8]. We identified bariatric procedures using the International Classification of Diseases, 9th Revision, Clinical Modification (ICD-9-CM) codes, Current Procedure Terminology codes (CPT-4), and Healthcare Common Procedure Coding System (HCPCS) codes (list available from authors by request). There were relatively few additional eligibility criteria (Figure 1) in order to maximize the representativeness of the cohort.

We extracted information on patient demographics (eg, age, sex, race/ethnicity), height, weight, BMI, blood pressure, and select comorbidities (eg, diabetes, sleep apnea) from the standardized data domains described in Table 1. Comorbidities were identified by ICD-9-CM diagnosis and procedure codes and the Systematized Nomenclature of Medicine (SNOMED) codes. We also calculated a combined comorbidity score that merges the Charlson and Elixhauser comorbidity scores [9]. The score, calculated based on 20 conditions identified by ICD-9-CM and SNOMED codes in the year prior to surgery, was initially developed to predict mortality. It has been shown to be a good proxy for general health status and has been used in prior analysis of bariatric patients [10].

### Follow-Up

Patients were followed as part of routine clinical care in each participating health system. We used BMI measurements after the index bariatric procedure as a proxy for follow-up. Because the United States transitioned from the ICD-9-CM coding system to the ICD-10-CM system on October 1, 2015, we ended follow-up on September 30, 2015 to avoid changes related to coding of diagnoses and procedures.

### Analysis

The baseline characteristics of the study cohort were compared by procedure type. The temporal trends in bariatric procedures during the study period were also assessed. Within the study cohort, the characteristics of patients with and without a BMI measurement during follow-up were further compared. We also examined the length of follow-up by procedure type. Finally, we compared the study cohort with patients who were excluded from the study due to missing baseline BMI measurement. We performed all the comparisons separately for the adult and adolescent subcohorts.

### Stakeholder Engagement

In addition to the extensive clinical data and research infrastructure necessary to collect the data for the study, a unique aspect of the PCORnet Bariatric Study is the engagement of a broad range of stakeholders. As part of our initial work to formulate the proposal, we identified 4 key stakeholder groups that would be critical to the success of our project: patients and caregivers, healthcare providers, healthcare system or organizational leaders, and community and advocacy groups. Each participating network was asked to engage a stakeholder as part of their research team and representatives from each of these groups formed an Executive Stakeholder Advisory Board and advised the scientific investigators on all aspects of the conduct of the study.

## Results

### Baseline Characteristics of the Study Cohort

#### Adult Subcohort

There were 65,093 adult bariatric patients in the PBS cohort, more than 10 times the size of the well-established Longitudinal Assessment of Bariatric Surgery study cohort [11]. These adult patients had a mean age of 45 years and were predominantly female (79.30%, 51,619/65,093) (Table 2). Among patients with non-missing race or ethnicity information, 72.08% (41,248/57,227) were White, 21.13% (12,094/57,227) were Black, and 20.58% (13,094/63,637) had an ethnicity of Hispanic recorded in their EHRs. The mean maximum BMI at baseline was 49 kg/m^2^, with 37.26% (24,255/65,093) having a BMI measurement of 50 kg/m^2^ or more. More than half of the patients (58.83%, 38,297/65,093) had a hypertension diagnosis in their EHRs. Other common conditions recorded in the EHR included sleep apnea (48.83%, 31,785/65,093), dyslipidemia (46.67%, 30,377/65,093), gastroesophageal reflux disease (GERD) (40.94%, 26,650/65,093), diabetes (35.97%, 23,411/65,093), depression (29.69%, 19,324/65,093), anxiety (20.98%, 13,657/65,093), and non-alcoholic fatty liver disease (NAFLD) (20.42%, 13,293/65,093).

RYGB was the most common bariatric procedure in this subcohort (49.48%, 32,208/65,093), followed by SG (45.62%, 29,693/65,093) and AGB (4.90%, 3192/65,093) (Table 2). The SG patients appeared to be slightly younger at the time of surgery while the AGB patients had a lower mean maximum baseline BMI. The frequency of numerous comorbidities differed by procedure type, with RYGB patients typically showing higher prevalence of pre-operative comorbidity than patients with other procedure types. There was racial and ethnic variation in the type of procedures received—the proportion of Black patients ranged from 15.65% (4515/28,845) in the RYGB group to 27.05% (6894/25,485) in the SG group, and the proportion of Hispanic patients ranged from 11.53% (352/3,054) in the AGB group to 24.58% (7144/29,059) in the RYGB group.

**Table 2:** Baseline^a^ characteristics of adult patients aged 20-79 years in the PCORnet Bariatric Study cohort (N=65,093).

Characteristic		AGB^b^	RYGB^c^	SG^d^	All
Number, (%)		3192 (100.00)	32,208 (100.00)	29,693 (100.00)	65,093 (100.00)
Age at surgery, mean (SD)		45.7 (12.35)	45.5 (11.63)	44.3 (11.72)	45.0 (11.72)
**Age category, n (%)**					
	20-44 years	1509 (47.27)	15,146 (47.03)	15,481 (52.14)	32,136 (49.37)
	45-64 years	1460 (45.74)	15,357 (47.68)	12,805 (43.12)	29,622 (45.51)
	65-79 years	223 (6.99)	1705 (5.29)	1407 (4.74)	3335 (5.12)
Female, n (%)		2524 (79.07)	25,679 (79.73)	23,416 (78.86)	51,619 (79.30)
Baseline maximum BMI, mean (SD)		46.1 (6.80)	49.5 (8.23)	48.8 (8.29)	49.0 (8.23)
**Baseline maximum BMI category, n (%)**					
	35-39 kg/m^2^	547 (17.14)	2566 (7.97)	2715 (9.14)	5828 (8.95)
	40-49 kg/m^2^	1897 (59.43)	16,740 (51.97)	16,373 (55.14)	35,010 (53.78)
	50-59 kg/m^2^	623 (19.52)	9435 (29.29)	7641 (25.73)	17,699 (27.19)
	≥60 kg/m^2^	125 (3.92)	3467 (10.76)	2964 (9.98)	6556 (10.07)
**Year of surgery, n (%)**					
	2005-2009	572 (17.92)	4519 (14.03)	481 (1.62)	5572 (8.56)
	2010	719 (22.53)	4407 (13.68)	1368 (4.61)	6494 (9.98)
	2011	785 (24.59)	5735 (17.81)	3772 (12.70)	10,292 (15.81)
	2012	591 (18.52)	5364 (16.65)	4930 (16.60)	10,885 (16.72)
	2013	300 (9.40)	4703 (14.60)	6118 (20.60)	11,121 (17.08)
	2014	175 (5.48)	4414 (13.70)	7394 (24.90)	11,983 (18.41)
	2015	50 (1.57)	3066 (9.52)	5630 (18.96)	8746 (13.44)
Hispanic^f^, n (%)		352 (11.53)	5598 (17.76)	7144 (24.58)	13,094 (20.58)
**Race category^f^, n (%)**					
	American Indian or Alaska Native	13 (0.45)	186 (0.64)	155 (0.61)	354 (0.62)
	Asian	15 (0.52)	225 (0.78)	300 (1.18)	540 (0.94)
	Black or African American	685 (23.65)	4515 (15.65)	6894 (27.05)	12,094 (21.13)
	Native Hawaiian or Other Pacific Islander	2 (0.07)	98 (0.34)	88 (0.35)	188 (0.33)
	White	2031 (70.11)	22,628 (78.45)	16,589 (65.09)	41,248 (72.08)
	Multiple race	43 (1.48)	438 (1.52)	321 (1.26)	802 (1.40)
	Other	108 (3.73)	755 (2.62)	1138 (4.47)	2001 (3.50)
Missing race, n (%)		295 (9.24)	3363 (10.44)	4208 (14.17)	7866 (12.08)
Systolic BP^g^, mean (SD)		128.1 (16.46)	131.0 (18.54)	131.6 (17.26)	131.1 (17.89)
Diastolic BP, mean (SD)		76.7 (11.24)	76.0 (12.43)	75.4 (11.75)	75.8 (12.08)
Missing BP, n (%)		240 (7.52)	1930 (5.99)	1836 (6.18)	4006 (6.15)
**Combined comorbidity score^h^, n (%)**					
	<0	1022 (32.02)	10,310 (32.01)	8911 (30.01)	20,243 (31.10)
	0	1805 (56.55)	16,672 (51.76)	16,675 (56.16)	35,152 (54.00)
	>0	365 (11.43)	5226 (16.23)	4107 (13.83)	9698 (14.90)
No. of hospital days in year before surgery, mean (SD)		0.30 (2.22)	0.46 (4.61)	0.47 (4.28)	0.46 (4.37)
**Health conditions^i^, n (%)**					
	Anxiety	557 (17.45)	7105 (22.06)	5995 (20.19)	13,657 (20.98)
	Depression	799 (25.03)	10,451 (32.45)	8074 (27.19)	19,324 (29.69)
	Diabetes	942 (29.51)	13,845 (42.99)	8624 (29.04)	23,411 (35.97)
	DVT^j^	20 (0.63)	220 (0.68)	214 (0.72)	454 (0.70)
	Dyslipidemia	1373 (43.01)	16,251 (50.46)	12,753 (42.95)	30,377 (46.67)
	Eating disorder	158 (4.95)	4441 (13.79)	1722 (5.80)	6321 (9.71)
	GERD^k^	1171 (36.69)	14726 (45.72)	10,753 (36.21)	26,650 (40.94)
	Hypertension	1820 (57.02)	20,210 (62.75)	16,267 (54.78)	38,297 (58.83)
	Infertility	20 (0.63)	215 (0.67)	213 (0.72)	448 (0.69)
	Kidney disease	169 (5.29)	2764 (8.58)	1964 (6.62)	4897 (7.52)
	NAFLD^l^	429 (13.44)	8148 (25.30)	4716 (15.88)	13,293 (20.42)
	Osteoarthritis	58 (1.82)	585 (1.82)	577 (1.94)	1220 (1.87)
	PCOS^m^	141 (4.42)	1741 (5.41)	1463 (4.93)	3345 (5.14)
	PE^n^	29 (0.91)	402 (1.25)	286 (0.96)	717 (1.10)
	Psychotic disorder	70 (2.19)	1098 (3.41)	822 (2.77)	1990 (3.06)
	Sleep apnea	1362 (42.67)	17,583 (54.59)	12,840 (43.24)	31,785 (48.83)
	Smoking	185 (5.80)	2987 (9.27)	2380 (8.02)	5552 (8.53)
	Substance use disorder	31 (0.97)	673 (2.09)	615 (2.07)	1319 (2.03)

^a^Baseline: identified in the year prior to surgery.

^b^AGB: adjustable gastric banding.

^c^RYGB: Roux-en-Y gastric bypass.

^d^SG: sleeve gastrectomy.

^e^BMI: body mass index.

^f^Number and proportion are calculated among patients with non-missing race (or ethnicity) information.

^g^BP: blood pressure.

^h^The combined comorbidity score merges the Charlson and Elixhauser comorbidity scores [9]. It is calculated based on 20 conditions identified by ICD-9-CM and SNOMED codes in the year prior to surgery. The score ranges from –2 to 26, with a higher score generally indicating poorer health status.

^i^Identified by one or more ICD-9-CM or SNOMED diagnosis code in the year prior to surgery.

^j^DVT: deep vein thrombosis.

^k^GERD: gastroesophageal reflux disease.

^l^NAFLD: non-alcoholic fatty liver disease.

^m^PCOS: polycystic ovarian syndrome.

^n^PE: pulmonary embolism.

#### Adolescent Subcohort

The PBS cohort also included 777 adolescent bariatric patients, more than twice the size of the largest published bariatric study of an adolescent population (Table 3) [12]. The mean age of the adolescent subcohort was 17 years and 77.5% (602/777) were female. Among patients with race or ethnicity information, 67.3% (473/703) were White, 22.6% (159/703) were Black, and 18.0% (124/689) were Hispanic. The mean maximum BMI at baseline was 53 kg/m^2^, with 54.3% (422/777) having a BMI measurement of 50 kg/m^2^ or more. As in the adult subcohort, the prevalence of having certain comorbid conditions recorded in the EHR was high in the adolescent patients. Sleep apnea (37.1%, 288/777), dyslipidemia (34.0%, 264/777), and hypertension (30.5%, 237/777) each occurred in more than 30% of the adolescent patients. GERD (26.1%, 203/777), depression (26.1%, 203/777), polycystic ovarian syndrome (PCOS) (20.9%, 162/777), NAFLD (19.7%, 153/777), anxiety (17.3%, 134/777), and diabetes (15.7%, 122/777) were also common. The majority of the adolescent patients received SG (60.4%, 469/777), followed by RYGB (30.8%, 239/777) and AGB (8.9%, 69/777). The mean age and baseline BMI were quite similar across the three treatment groups. The RYGB patients appeared to have more comorbid conditions recorded in their EHRs than the other 2 groups, but these prevalence estimates may be less reliable than in the adult subcohort.

**Table 3:** Baseline^a^ characteristics of adolescent patients aged 12 to 19 years in the PCORnet Bariatric Study cohort (N=777).

Characteristic		AGB^b^	RYGB^c^	SG^d^	All
Number, (%)		69 (100.0)	239 (100.0)	469 (100.0)	777 (100.0)
Age at surgery, mean (SD)		17.4 (1.3)	17.7 (1.4)	17.3 (1.6)	17.5 (1.5)
**Age category, n (%)**					
	12 years	0 (0.0)	0 (0.0)	2 (0.4)	2 (0.3)
	13-15 years	6 (8.7)	18 (7.5)	68 (14.5)	92 (11.8)
	16-17 years	28 (40.6)	56 (23.4)	136 (29.0)	220 (28.3)
	18-19 years	35 (50.7)	165 (69.0)	263 (56.1)	463 (59.6)
Female, n (%)		55 (79.7)	191 (79.9)	356 (75.9)	602 (77.5)
Baseline maximum BMI, mean (SD)		51.4 (7.9)	53.2 (9.1)	52.6 (9.0)	52.7 (8.9)
**Baseline maximum BMI category, n (%)**					
	35-39 kg/m^2^	1 (1.5)	5 (2.1)	4 (0.9)	10 (1.3)
	40-49 kg/m^2^	34 (49.3)	99 (41.4)	212 (45.2)	345 (44.4)
	50-59 kg/m^2^	28 (40.6)	88 (36.8)	163 (34.8)	279 (35.9)
	≥60 kg/m^2^	6 (8.7)	47 (19.7)	90 (19.2)	143 (18.4)
**Year of surgery, n (%)**					
	2005-2009	21 (30.4)	28 (11.7)	8 (1.7)	57 (7.3)
	2010	17 (24.6)	32 (13.4)	36 (7.7)	85 (10.9)
	2011	11 (15.9)	50 (20.9)	50 (10.7)	111 (14.3)
	2012	10 (14.5)	35 (14.6)	83 (17.7)	128 (16.5)
	2013	9 (13.0)	42 (17.6)	92 (19.6)	143 (18.4)
	2014	1 (1.5)	28 (11.7)	124 (26.4)	153 (19.7)
	2015	0 (0.0)	24 (10.0)	76 (16.2)	100 (12.9)
Hispanic^f^, n (%)		4 (6.5)	43 (22.3)	77 (17.7)	124 (18.0)
**Race category^f^, n (%)**					
	American Indian or Alaska Native	0 (0.0)	1 (0.5)	4 (1.0)	5 (0.7)
	Asian	0 (0.0)	2 (0.9)	3 (0.7)	5 (0.7)
	Black or African American	11 (16.7)	38 (17.5)	110 (26.2)	159 (22.6)
	Native Hawaiian or Other Pacific Islander	0 (0.0)	0 (0.0)	0 (0.0)	0 (0.0)
	White	45 (68.2)	160 (73.7)	268 (63.8)	473 (67.3)
	Multiple race	3 (4.6)	8 (3.7)	15 (3.6)	26 (3.7)
	Other	7 (10.6)	8 (3.7)	20 (4.8)	35 (5.0)
Missing race, n (%)		3 (4.4)	22 (9.2)	49 (10.5)	74 (9.5)
Systolic BP^g^, mean (SD)		125.9 (16.5)	128.6 (15.9)	130.2 (17.0)	129.3 (16.6)
Diastolic BP, mean (SD)		75.2 (11.5)	74.1 (12.2)	70.3 (12.1)	71.9 (12.2)
Missing BP, n (%)		1 (1.5)	13 (5.4)	14 (3.0)	28 (3.6)
**Combined comorbidity score^h^, n (%)**					
	<0	1 (1.5)	20 (8.4)	46 (9.8)	67 (8.6)
	0	66 (95.7)	148 (61.9)	324 (69.1)	538 (69.2)
	>0	2 (2.9)	71 (29.7)	99 (21.1)	172 (22.1)
No. of hospital days in year before surgery, mean (SD)		1.5 (4.4)	0.2 (1.9)	2.4 (12.6)	1.7 (10.0)
**Health conditions^i^, n (%)**					
	Anxiety	11 (15.9)	44 (18.4)	79 (16.8)	134 (17.3)
	Depression	22 (31.9)	68 (28.5)	113 (24.1)	203 (26.1)
	Diabetes	5 (7.3)	52 (21.8)	65 (13.9)	122 (15.7)
	DVT^j^	0 (0.0)	0 (0.0)	0 (0.0)	0 (0.0)
	Dyslipidemia	30 (43.5)	71 (29.7)	163 (34.8)	264 (34.0)
	Eating disorder	1 (1.5)	23 (9.6)	14 (3.0)	38 (4.9)
	GERD^k^	12 (17.4)	74 (31.0)	117 (25.0)	203 (26.1)
	Hypertension	29 (42.0)	70 (29.3)	138 (29.4)	237 (30.5)
	Infertility	0 (0.0)	4 (1.7)	7 (1.5)	11 (1.4)
	Kidney disease	0 (0.0)	3 (1.3)	6 (1.3)	9 (1.2)
	NAFLD^l^	2 (2.9)	81 (33.9)	70 (14.9)	153 (19.7)
	Osteoarthritis	0 (0.0)	0 (0.0)	0 (0.0)	0 (0.0)
	PCOS^m^	12 (17.4)	61 (25.5)	89 (19.0)	162 (20.9)
	PE^n^	0 (0.0)	0 (0.0)	1 (0.2)	1 (0.1)
	Psychotic disorder	1 (1.5)	7 (2.9)	12 (2.6)	20 (2.6)
	Sleep apnea	11 (15.9)	113 (47.3)	164 (35.0)	288 (37.1)
	Smoker	1 (1.5)	11 (4.6)	23 (4.9)	35 (4.5)
	Substance use disorder	1 (1.5)	1 (0.4)	2 (0.4)	4 (0.5)

^a^Baseline: identified in the year prior to surgery.

^b^AGB: adjustable gastric banding.

^c^RYGB: Roux-en-Y gastric bypass.

^d^SG: sleeve gastrectomy.

^e^BMI: body mass index.

^f^Number and proportion are calculated among patients with non-missing race (or ethnicity) information.

^g^BP: blood pressure.

^h^The combined comorbidity score merges the Charlson and Elixhauser comorbidity scores [9]. It is calculated based on 20 conditions identified by ICD-9-CM and SNOMED codes in the year prior to surgery. The score ranges from –2 to 26, with a higher score generally indicating poorer health status.

^i^Identified by one or more ICD-9-CM or SNOMED diagnosis code in the year prior to surgery.

^j^DVT: deep vein thrombosis.

^k^GERD: gastroesophageal reflux disease.

^l^NAFLD: non-alcoholic fatty liver disease.

^m^PCOS: polycystic ovarian syndrome.

^n^PE: pulmonary embolism.

### Temporal Trends in Bariatric Procedures Performed in the Study Cohort

**Figure 2:**
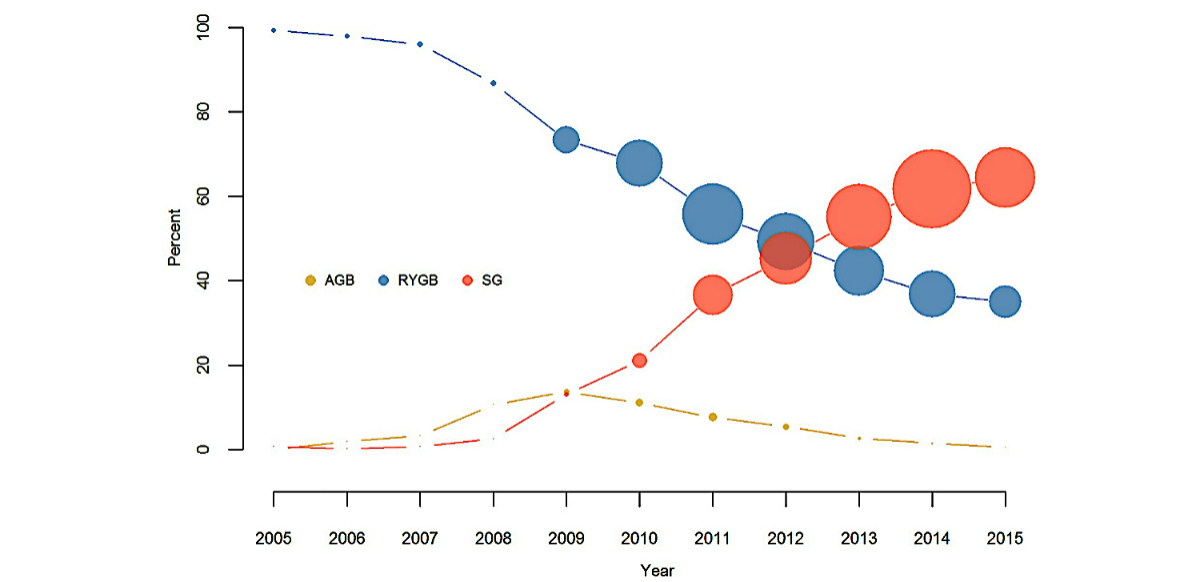
Shift in choice of bariatric procedure in adults in the PCORnet Bariatric Study from 2005 to 2015.Size of the data point is proportionate to the number of patients at that time point. All 11 Clinical Data Research Network (CDRNs) that participate in the study contribute data to all study years, but not all 42 participating health systems have data in all years. AGB: adjustable gastric banding; RYGB: Roux-en-Y gastric bypass; SG: sleeve gastrectomy.

**Figure 3:**
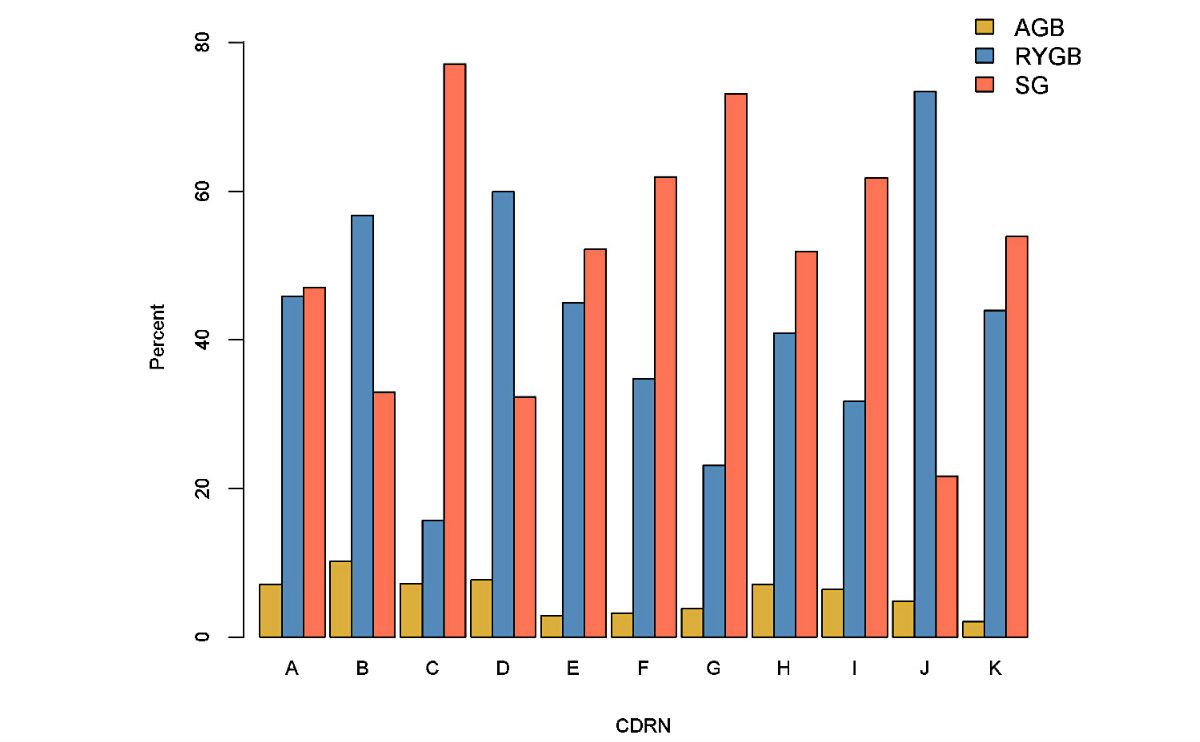
Variability in use of the three most common bariatric procedure types in adults in the PCORnet Bariatric Study, by Clinical Data Research Network (CDRN), 2005-2015. AGB: adjustable gastric banding; RYGB: Roux-en-Y gastric bypass; SG: sleeve gastrectomy.

We observed dramatic shifts in the type of procedures performed in adults between 2005 and 2015 (Figure 2). Almost all of the bariatric procedures performed in the health systems contributing to the dataset in 2005 were RYGB. SG became increasingly popular starting in 2010 and was the most commonly performed bariatric procedure by 2013. Although the shifts in the type of procedures performed in the adult subcohort are consistent with other studies, it is worth noting that while all 11 participating CDRNs contribute data to all study years, not all 42 participating health systems within these CDRNs have data in all years. We also found substantial variability in the type of procedures performed in adults across CDRNs during the study period (Figure 3). RYGB was the most commonly performed procedure in 3 CDRNs while SG was the primary procedure in 8 CDRNs. The proportion of RYGB procedure ranged from 16% to 69% across CDRNs.

**Table 4:** Characteristics of adult patients aged 20 to 79 years with or without a body mass index measurement in the electronic health record at baseline^a^ and in follow-up.

Characteristic		The Adult PCORnet Bariatric Study cohort			
		All	Patients with BMI^b^≥35 at baseline and a BMI measurement during follow-up	Patients with BMI ≥35 at baseline but missing BMI during follow-up	Patients with missing BMI at baseline (excluded from the study cohort)
Number (%)		65,093 (100.00)	46,510 (100.00)	18,583 (100.00)	12,510 (100.00)
**Bariatric procedure, n (%)**					
	AGB^c^	3192 (4.90)	2567 (5.52)	625 (3.36)	3049 (24.37)
	RYGB^d^	32,208 (49.48)	24,982 (53.71)	7226 (38.89)	6621 (52.93)
	SG^e^	29,693 (45.62)	18,961 (40.77)	10,732 (57.75)	2840 (22.70)
Age at surgery, mean (SD)		45.0 (11.72)	45.5 (11.60)	43.6 (11.91)	45.8 (12.10)
**Age category, n (%)**					
	20-44 years	32,136 (49.37)	22,075 (47.46)	10,061 (54.14)	5866 (46.89)
	45-64 years	29,622 (45.51)	22,042 (47.39)	7580 (40.79)	5782 (46.22)
	65-79 years	3335 (5.12)	2393 (5.15)	942 (5.07)	862 (6.89)
Female, n (%)		51,619 (79.30)	37,315 (80.23)	14,304 (76.97)	9535 (76.23)
Baseline maximum BMI, mean (SD)		49.0 (8.23)	49.1 (8.17)	48.8 (8.39)	N/A
**Baseline maximum BMI category, n (%)**					
	35-39 kg/m^2^	5828 (8.95)	3865 (8.31)	1963 (10.56)	0 (0.0)
	40-49 kg/m^2^	35,010 (53.78)	25,123 (54.02)	9887 (53.20)	0 (0.0)
	50-59 kg/m^2^	17,699 (27.19)	12,870 (27.67)	4829 (25.99)	0 (0.0)
	≥60 kg/m^2^	6556 (10.07)	4652 (10.00)	1904 (10.25)	0 (0.0)
	Missing	0 (0.0)	0 (0.0)	0 (0.0)	12,510 (100.00)
**Year of surgery, n (%)**					
	2005-2009	5572 (8.56)	5183 (11.14)	389 (2.09)	2532 (20.24)
	2010	6494 (9.98)	5819 (12.51)	675 (3.63)	2721 (21.75)
	2011	10,292 (15.81)	9015 (19.38)	1277 (6.87)	2518 (20.13)
	2012	10,885 (16.72)	8977 (19.30)	1908 (10.27)	2041 (16.31)
	2013	11,121 (17.08)	8556 (18.40)	2565 (13.80)	1227 (9.81)
	2014	11,983 (18.41)	7951 (17.10)	4032 (21.70)	896 (7.16)
	2015	8746 (13.44)	1009 (2.17)	7737 (41.63)	575 (4.60)
Hispanic^f^, n (%)		13,094 (20.58)	9612 (21)	3482 (19.49)	876 (8.00)
**Race category^f^, n (%)**					
	American Indian or Alaska Native	354 (0.62)	279 (0.68)	75 (0.46)	45 (0.42)
	Asian	540 (0.94)	422 (1.03)	118 (0.73)	63 (0.59)
	Black or African American	12,094 (21.13)	8526 (20.76)	3568 (22.09)	1586 (14.86)
	Native Hawaiian or Other Pacific Islander	188 (0.33)	143 (0.35)	45 (0.28)	9 (0.08)
	White	41,248 (72.08)	30,371 (73.95)	10,877 (67.33)	8523 (79.87)
	Multiple race	802 (1.40)	465 (1.13)	337 (2.09)	90 (0.84)
	Other	2001 (3.50)	866 (2.11)	1135 (7.03)	355 (3.33)
Missing race, n (%)		7866 (12.08)	5438 (11.69)	2428 (13.07)	1839 (14.70)
Systolic BP^g^, mean (SD)		131.1 (17.89)	130.5 (17.08)	132.8 (19.78)	131.1 (16.69)
Diastolic BP, mean (SD)		75.8 (12.08)	75.6 (11.56)	76.1 (13.36)	76.1 (11.41)
Missing BP, n (%)		4006 (6.15)	1801 (3.87)	2205 (11.87)	10,736 (85.82)
**Combined comorbidity score^h^, n (%)**					
	<0	20,243 (31.10)	15,488 (33.30)	4755 (25.59)	2845 (22.74)
	0	35,152 (54.00)	23,879 (51.34)	11,273 (60.66)	8463 (67.65)
	>0	9698 (14.90)	7143 (15.36)	2555 (13.75)	1202 (9.61)
No. of hospital days in year before surgery, mean (SD)		0.46 (4.37)	0.43 (3.74)	0.53 (5.66)	0.79 (9.52)
**Health conditions^i^, n (%)**					
	Anxiety	13,657 (20.98)	9917 (21.32)	3740 (20.13)	1837 (14.68)
	Depression	19,324 (29.69)	14,339 (30.83)	4985 (26.83)	3370 (26.94)
	Diabetes	23,411 (35.97)	17,320 (37.24)	6091 (32.78)	3835 (30.66)
	DVT^j^	454 (0.70)	336 (0.72)	118 (0.63)	94 (0.75)
	Dyslipidemia	30,377 (46.67)	22,823 (49.07)	7554 (40.65)	5152 (41.18)
	Eating disorder	6321 (9.71)	4983 (10.71)	1338 (7.20)	238 (1.90)
	GERD^k^	26,650 (40.94)	18,995 (40.84)	7655 (41.19)	4764 (38.08)
	Hypertension	38,297 (58.83)	28,017 (60.24)	10,280 (55.32)	7089 (56.67)
	Infertility	448 (0.69)	339 (0.73)	109 (0.59)	75 (0.60)
	Kidney disease	4897 (7.52)	3824 (8.22)	1073 (5.77)	491 (3.92)
	NAFLD^l^	13,293 (20.42)	9646 (20.74)	3647 (19.63)	1380 (11.03)
	Osteoarthritis	1220 (1.87)	814 (1.75)	406 (2.18)	165 (1.32)
	PCOS^m^	3345 (5.14)	2345 (5.04)	1000 (5.38)	435 (3.48)
	PE^n^	717 (1.10)	547 (1.18)	170 (0.91)	96 (0.77)
	Psychotic disorder	1990 (3.06)	1471 (3.16)	519 (2.79)	235 (1.88)
	Sleep apnea	31,785 (48.83)	22,894 (49.22)	8891 (47.84)	5254 (42.00)
	Smoking	5552 (8.53)	4008 (8.62)	1544 (8.31)	618 (4.94)
	Substance use disorder	1319 (2.03)	984 (2.12)	335 (1.80)	199 (1.59)

^a^Baseline: identified in the year prior to surgery.

^b^BMI: body mass index.

^c^AGB: adjustable gastric banding.

^d^RYGB: Roux-en-Y gastric bypass.

^e^SG: sleeve gastrectomy.

^f^Number and proportion are calculated among patients with non-missing race (or ethnicity) information.

^g^BP: blood pressure.

^h^The combined comorbidity score merges the Charlson and Elixhauser comorbidity scores [9]. It is calculated based on 20 conditions identified by ICD-9-CM and SNOMED codes in the year prior to surgery. The score ranges from –2 to 26, with a higher score generally indicating poorer health status.

^i^Identified by one or more ICD-9-CM or SNOMED diagnosis code in the year prior to surgery.

^j^DVT: deep vein thrombosis.

^k^GERD: gastroesophageal reflux disease.

^l^NAFLD: non-alcoholic fatty liver disease.

^m^PCOS: polycystic ovarian syndrome.

^n^PE: pulmonary embolism.

### Comparisons Between Study Cohort and Patients Excluded From the Study

#### Adult Subcohort

We excluded 12,510 adult bariatric patients with missing baseline BMI in the EHR who met the other eligibility criteria of the study and an additional 1918 patients whose baseline BMI were less than 35 kg/m^2^. Compared to the 65,093 adult patients in the PBS cohort, patients with missing baseline BMI information were more often White (79.87%, 8523/10,671 versus 72.08%, 41,248/57,227), less often Black (14.86%, 1586/10,671 versus 21.13%, 12,094/57,227), less often Hispanic (8.00%, 876/10,951 versus 20.58%, 13,094/63,637), and more likely to have their procedure performed in earlier study years (Table 4). Fewer of the excluded patients had comorbid health conditions recorded in the EHR, such as anxiety (14.68%, 1837/12,510 versus 20.98%, 13,657/65,093), eating disorder (1.90%, 238/12,510 versus 9.71%, 6321/65,093), NAFLD (11.03%, 1380/12,510 versus 20.42%, 13,293/65,093), and sleep apnea (42.00%, 5254/12,510 versus 48.83%, 31,785/65,093). Not surprisingly, patients without a baseline BMI measurement also had a much higher proportion of missing blood pressure measurements (85.82%, 10,736/12,510 versus 6.15%, 4006/65,093). Ongoing and future analyses will account for the differences in the patient characteristics.

#### Adolescent Subcohort

We excluded 127 adolescent patients with missing baseline BMI in the EHR who met the other eligibility criteria of the study and an additional 54 patients whose baseline BMI were less than 35 kg/m^2^. Compared to the adolescent patients in the PBS cohort, patients with missing baseline BMI information were more likely to have undergone AGB or RYGB procedure and more likely to have their bariatric procedures performed in earlier study years (Table 5). They were also less often female (64.6%, 82/127 versus 77.5%, 602/777), and had lower proportions of comorbid conditions recorded in the EHR, such as depression (15.8%, 20/127 versus 26.1%, 203/777), diabetes (4.7%, 6/127 versus 15.7%, 122/777), dyslipidemia (8.7%, 11/127 versus 34.0%, 264/777), hypertension (12.6%, 16/127 versus 30.5%, 237/777), NAFLD (6.3%, 8/127 versus 19.7%, 153/777), PCOS (5.5%, 7/127 versus 20.9%, 162/777), and sleep apnea (16.5%, 21/127 versus 37.1%, 288/777). The excluded patients spent more days in the hospital, on average, in the year prior to the surgery compared to those in the PBS cohort (6 versus 2 days).

### Follow-Up

#### Adult Subcohort

Within the adult subcohort, 71.45% (46,510/65,093) patients had one or more BMI measurements beyond 6 months of post-operative follow-up. However, follow-up ended on September 30, 2015, so not all patients were eligible to be followed for 1, 3, or 5 full years. For example, only patients who had a bariatric procedure on October 1, 2010 or earlier could be followed for 5 complete years during the study’s timeframe. The proportion of eligible patients with at least one BMI measurement in the follow-up windows of interest was 84.31% (44,978/53,351) at 6 to 18 months, 68.09% (20,783/30,521) at 30 to 42 months, and 68.56% (7159/10,442) at 54 to 66 months after surgery (Table 6). Long-term follow-up varied by treatment group, with SG patients being most likely to have a BMI measurement at years 3 and 5, followed by RYGB patients and AGB patients.

**Table 5:** Characteristics of adolescent patients aged 12 to 19 years with or without a body mass index measurement in the electronic health record at baseline^a^ and in follow-up.

Characteristic		The Adolescent PCORnet Bariatric Study cohort			
		All	Patients with BMI^b^≥35 at baseline and a BMI measurement during follow-up	Patients with BMI ≥35 at baseline but missing BMI during follow-up	Patients with missing BMI at baseline (excluded from the study cohort)
Number (%)		777 (100.0)	544 (100.0)	233 (100.0)	127 (100.0)
**Bariatric procedure, n (%)**					
	AGB^c^	69 (8.9)	61 (11.2)	8 (3.4)	36 (28.4)
	RYGB^d^	239 (30.8)	177 (32.5)	62 (26.6)	62 (48.8)
	SG^e^	469 (60.4)	306 (56.3)	163 (70.0)	29 (22.8)
Age at surgery, mean (SD)		17.5 (1.5)	17.3 (1.6)	17.7 (1.3)	17.7 (1.6)
**Age category, n (%)**					
	12 years	2 (0.3)	2 (0.4)	0 (0.0)	3 (2.4)
	13-15 years	92 (11.8)	75 (13.8)	17 (7.3)	11 (8.7)
	16-17 years	220 (28.3)	157 (28.9)	63 (27.0)	28 (22.1)
	18-19 years	463 (59.6)	310 (57.0)	153 (65.7)	85 (66.9)
Female, n (%)		602 (77.5)	428 (78.7)	174 (74.7)	82 (64.6)
Baseline maximum BMI, mean (SD)		52.7 (8.9)	52.5 (8.5)	53.0 (9.9)	N/A
**Baseline maximum BMI category, n (%)**					
	35-39 kg/m^2^	10 (1.3)	5 (0.9)	5 (2.2)	0 (0.0)
	40-49 kg/m^2^	345 (44.4)	249 (45.8)	96 (41.2)	0 (0.0)
	50-59 kg/m^2^	279 (35.9)	192 (35.3)	87 (37.3)	0 (0.0)
	≥60 kg/m^2^	143 (18.4)	98 (18.0)	45 (19.3)	0 (0.0)
	Missing	0 (0.0)	0 (0.0)	0 (0.0)	127 (100.0)
**Year of surgery, n (%)**					
	2005-2009	57 (7.3)	52 (9.6)	5 (2.2)	34 (26.8)
	2010	85 (10.9)	78 (14.3)	7 (3.0)	18 (14.2)
	2011	111 (14.3)	98 (18.0)	13 (5.6)	24 (18.9)
	2012	128 (16.5)	106 (19.5)	22 (9.4)	31 (24.4)
	2013	143 (18.4)	105 (19.3)	38 (16.3)	10 (7.9)
	2014	153 (19.7)	101 (18.6)	52 (22.3)	6 (4.7)
	2015	100 (12.9)	4 (0.7)	96 (41.2)	4 (3.2)
Hispanic^f^, n (%)		124 (18.0)	81 (16.8)	43 (20.8)	14 (18.9)
**Race category^f^, n (%)**					
	American Indian or Alaska Native	5 (0.7)	3 (0.6)	2 (1.0)	2 (1.8)
	Asian	5 (0.7)	5 (1.0)	0 (0.0)	1 (0.9)
	Black or African American	159 (22.6)	122 (24.6)	37 (18.0)	26 (23.6)
	Native Hawaiian or Other Pacific Islander	0 (0.0)	0 (0.0)	0 (0.0)	0 (0.0)
	White	473 (67.3)	330 (66.4)	143 (69.4)	74 (67.3)
	Multiple race	26 (3.7)	12 (2.4)	14 (6.8)	1 (0.9)
	Other	35 (5.0)	25 (5.0)	10 (4.9)	6 (5.5)
Missing race, n (%)		74 (9.5)	47 (8.6)	27 (11.6)	17 (13.4)
Systolic BP^g^, mean (SD)		129.3 (16.6)	128.5 (16.7)	131.3 (16.4)	119.6 (16.3)
Diastolic BP, mean (SD)		71.9 (12.2)	71.3 (12.0)	73.3 (12.7)	68.5 (10.2)
Missing BP, n (%)		28 (3.6)	12 (2.2)	16 (6.9)	111 (87.4)
**Combined comorbidity score^h^, n (%)**					
	<0	67 (8.6)	46 (8.5)	21 (9.0)	2 (1.6)
	0	538 (69.2)	365 (67.1)	173 (74.3)	102 (80.3)
	>0	172 (22.1)	133 (24.5)	39 (16.7)	23 (18.1)
No. of hospital days in year before surgery, mean (SD)		1.7 (10.0)	1.5 (7.9)	2.1 (13.7)	6.4 (39.6)
**Health conditions^i^, n (%)**					
	Anxiety	134 (17.3)	87 (16.0)	47 (20.2)	18 (14.2)
	Depression	203 (26.1)	151 (27.8)	52 (22.3)	20 (15.8)
	Diabetes	122 (15.7)	88 (16.2)	34 (14.6)	6 (4.7)
	DVT^j^	0 (0.0)	0 (0.0)	0 (0.0)	1 (0.8)
	Dyslipidemia	264 (34.0)	192 (35.3)	72 (30.9)	11 (8.7)
	Eating disorder	38 (4.9)	25 (4.6)	13 (5.6)	7 (5.5)
	GERD^k^	203 (26.1)	137 (25.2)	66 (28.3)	29 (22.8)
	Hypertension	237 (30.5)	175 (32.2)	62 (26.6)	16 (12.6)
	Infertility	11 (1.4)	4 (0.7)	7 (3.0)	0 (0.0)
	Kidney disease	9 (1.2)	7 (1.3)	2 (0.9)	9 (7.1)
	NAFLD^l^	153 (19.7)	103 (18.9)	50 (21.5)	8 (6.3)
	Osteoarthritis	0 (0.0)	0 (0.0)	0 (0.0)	1 (0.8)
	PCOS^m^	162 (20.9)	120 (22.1)	42 (18.0)	7 (5.5)
	PE^n^	1 (0.1)	1 (0.2)	0 (0.0)	0 (0.0)
	Psychotic disorder	20 (2.6)	14 (2.6)	6 (2.6)	2 (1.6)
	Sleep apnea	288 (37.1)	198 (36.4)	90 (38.6)	21 (16.5)
	Smoking	35 (4.5)	25 (4.6)	10 (4.3)	4 (3.2)
	Substance use disorder	4 (0.5)	2 (0.4)	2 (0.9)	3 (2.4)

^a^Baseline: identified in the year prior to surgery.

^b^BMI: body mass index.

^c^AGB: adjustable gastric banding.

^d^RYGB: Roux-en-Y gastric bypass.

^e^SG: sleeve gastrectomy.

^f^Number and proportion are calculated among patients with non-missing race (or ethnicity) information.

^g^BP: blood pressure.

^h^The combined comorbidity score merges the Charlson and Elixhauser comorbidity scores [9]. It is calculated based on 20 conditions identified by ICD-9-CM and SNOMED codes in the year prior to surgery. The score ranges from –2 to 26, with a higher score generally indicating poorer health status.

^i^Identified by one or more ICD-9-CM or SNOMED diagnosis code in the year prior to surgery.

^j^DVT: deep vein thrombosis.

^k^GERD: gastroesophageal reflux disease.

^l^NAFLD: non-alcoholic fatty liver disease.

^m^PCOS: polycystic ovarian syndrome.

^n^PE: pulmonary embolism.

**Table 6:** Follow-up information in the PCORnet Bariatric Study cohort.

Cohort		Follow-up window of interest		
		1 year (measured at 6 to 18 months)	3 years (measured at 30 to 42 months)	5 years (measured at 54 to 66 months)
**Number and proportion of patients in the adult subcohort having a BMI^a^measurement during follow-up, among patients eligible^b^, n/N (%)**				
	All	44,978/53,351 (84.31%)	20,783/30,521 (68.09%)	7159/10,442 (68.56%)
	AGB^c^	2367/3098 (76.40%)	1507/2519 (59.82%)	609/1111 (54.82%)
	RYGB^d^	24,061/28,039 (85.81%)	12,429/18,684 (66.52%)	5257/7824 (67.19%)
	SG^e^	18,550/22,214 (83.50%)	6847/9318 (73.48%)	1293/1507 (85.79%)
**Number and proportion of patients in the adolescent subcohort** **having a BMI measurement during follow-up, among patients eligible^b^, n/N (%)**				
	All	524/639 (82.0%)	174/349 (49.9%)	47/121 (38.8%)
	AGB	58/69 (84.1%)	21/57 (36.8%)	6/34 (17.6%)
	RYGB	165/208 (79.3%)	69/136 (50.7%)	25/52 (48.1%)
	SG	301/362 (83.1%)	84/156 (53.8%)	16/35 (45.7%)

^a^BMI: body mass index.

^b^Number of patients who can be followed for a certain follow-up window of interest based on the study timeframe, which ended on September 30, 2015. For example, only patients who had a bariatric procedure on October 1, 2014 or earlier would be eligible for having one complete year of follow-up information. However, the number of eligible patients was an estimate because we did not request actual dates for the analysis for privacy consideration—all patients who had their procedure performed in 2013 or earlier and 3/4 of patients who had their procedure performed in 2014 will be eligible for at least one year of follow-up.

^c^AGB: adjustable gastric banding.

^d^RYGB: Roux-en-Y gastric bypass.

^e^SG: sleeve gastrectomy.

Among the adult patients in the PBS cohort, those without a BMI measurement during follow-up were overall quite similar to those with a measurement (Table 4). However, they appeared to be younger (44 versus 46 years), less often White (67.33%, 10,877/16,155 versus 73.95%, 30,371/41,072), and more likely to have their procedure performed in later study years. These patients generally have lower proportions of comorbid conditions, but the differences were relatively small.

#### Adolescent Subcohort

Within the adolescent subcohort, 70.0% (544/777) patients had at least 1 BMI measurement beyond 6 months of post-operative follow-up. Of eligible patients, 82.0% (524/639) had a BMI measurement at 6 to 18 months following their index procedure (Table 6). Weight data were available in 49.9% (174/349) of eligible patients at 30 to 42 months, and 38.9% (47/121) of eligible patients at 54 to 66 months. The proportion of patients with a BMI measurement at years 3 and 5 was lowest in the AGB group, and similar between the RYGB and SG groups.

Among the adolescent patients in the PBS cohort, those without a BMI measurement during follow-up were overall quite similar to those with a measurement (Table 5). However, they were less often Black (18.0%, 37/206 versus 24.6%, 122/497), more likely to have undergone SG (70.0%, 163/233 versus 56.3%, 306/544), have their procedures performed in later study years, and have lower prevalence of recorded depression (22.3%, 52/233 versus 27.8%, 151/544) and hypertension (26.6%, 62/233 versus 32.2%, 175/544) than those with a measurement.

## Discussion

### Principal Findings

In this large, population-based, retrospective cohort study using the national PCORnet data infrastructure, we have identified 65,093 adults and 777 adolescents who underwent 1 of the 3 most common bariatric procedures, AGB, RYGB, and SG, in 42 geographically diverse health systems. Over the time frame of the study (2005 to 2015), we observed a dramatic shift in bariatric procedure use (Figure 2), with a sharp decline in the proportions of patients undergoing RYGB and AGB and increase in the proportion undergoing SG. In particular, the large number of SG patients in this cohort (29,693 adults and 469 adolescents) makes this a valuable resource for comparative effectiveness research. We also observed heterogeneity in bariatric procedure preferences across the 11 participating CDRNs (Figure 3), which underscore the need for better comparative effectiveness research evidence to inform patient and provider decisions about bariatric surgery.

### Strengths

The ongoing PBS is one of the largest cohorts of patients with bariatric procedures in the United States. Patients are geographically and demographically diverse, which improves the generalizability of the research findings and allows examination of treatment effect heterogeneity. This, in turn, may result in findings that can more easily be applied to clinical decision-making. The ability to use real-world data collected as part of healthcare delivery not only allows us to collect long-term follow-up data efficiently and at a lower cost but also to learn from the routine practice of medicine.

A unique strength of the PBS study is the depth and diversity of its stakeholder involvement, which includes not only several patients as study team members, but also multiple pediatric and adult bariatric surgeons from different institutions, primary care and specialty physicians, researchers, and leaders of patient-level policy and advocacy organizations. Stakeholders are fully engaged in all stages of the protocol development, including formulating the research questions and the study aims, selecting outcomes that are of interest to the patients, and identifying methods to study these outcomes (eg, prioritization of variables for heterogeneity of treatment effect analyses). They are also actively involved in monitoring study conduct, interpreting data in the context of local patient populations and coding practices, and designing and implementing dissemination plans. This robust engagement strategy helps ensure that the products of this research study are meaningful to patients, clinicians, and policy makers.

By having sites translate source data into the CDM in PCORnet, researchers can distribute one query to all sites and receive back standardized output (eg, identical variable names and categories) from disparate data sources. Using the CDM avoids much of the redundant preparatory work that would otherwise be needed to assemble cohorts or count potential events and other endpoints. Code lists and query programs developed as part of this study can also be used for future studies that leverage the PCORnet CDM. The CDM and distributed data network framework has been shown to improve the efficiency of the conduct of multi-database studies [13-17].

The PBS employs an efficient ethical review process. Adherence to human subjects protections and regulations was addressed at the CDRN level. Some participating networks obtained Institutional Review Board (IRB) approval for the study’s protocol using an IRB reliance agreement across their sites; others created and relied on a central IRB [18]. At some CDRNs, individual site’s IRB determined that these analyses of de-identified data did not qualify as human subjects research. The Kaiser Permanente Washington Health Research Institute, the lead site of the PBS, obtained IRB approval for overseeing data collection and leading analyses.

### Ongoing and Planned Activities

Ongoing and planned investigations in the PBS include head-to-head comparisons of these procedures on long-term changes in weight, rates of diabetes remission and relapse, and incidences of major surgery-related adverse events. These comparisons will be conducted separately in adults and adolescents. Additional evaluations will examine the heterogeneity of treatment effects for important covariates such as age, sex, race, and comorbidities. Furthermore, selected analyses will compare pooled individual-level data analysis with more privacy-protecting analytic approaches that share less granular information [19,20].

Examination of mortality after bariatric surgery is challenging using only EHR data. Deaths are not typically captured in EHRs except if they occur during hospitalization or in the emergency room, or when a primary care provider becomes aware of a patient’s death and the information is entered manually into the EHR. Some sites within the participating CDRNs have linked to state or national death indices. The PBS plans to perform additional linkages to these death registries for a subset of the study population to increase the accuracy and completeness of death information. In addition, a number of pre-specified surgery-related adverse events, including re-hospitalization and re-operation after bariatric surgery, may be incompletely captured in EHRs because patients may get a portion of their care outside of the data-contributing health systems. The PBS study will link the EHR data from select health systems to insurance claims data to improve capture of these events.

### Limitations

This study has several limitations. A non-negligible number of bariatric patients had missing BMI data either at baseline or in follow-up, and the reasons for having missing measurements were generally not well-recorded during the study period. Because the PCORnet CDM typically reflects data stored as discrete data elements, it is possible that some EHR data (eg, BMI recorded in a clinician’s note instead of in the vital signs table) was not represented in our analyses. Long-term follow-up (eg, 5 years) information was not available in some patients. Relying primarily on routinely collected health data means our data collection process might not be as systematic as in other prospective cohort studies (eg, the Longitudinal Assessment of Bariatric Surgery study [11] and the Teen-Longitudinal Assessment of Bariatric Surgery study [12]). However, it does represent the information that informs patient and provider decisions in routine clinical care. There was also variability in data capture and documentation across health systems during the study period.

We did not validate the algorithms used to identify the comorbidities of interest (eg, sleep apnea). It is possible that these conditions were under-recorded or over-recorded in certain EHRs. However, the implementation of the PCORnet CDM helps standardize a core set of variables expected to be commonly used in research studies. There is currently no plan to conduct analyses using data beyond September 30, 2015. Although the PBS cohort will perform linkages with additional data sources to improve the completeness and accuracy of certain information, these linkages will not be performed in the entire cohort.

### Conclusion

Using the data and research infrastructure created by the PCORnet, we have created one of the largest cohorts of patients with bariatric procedures in the United States. The diversity of the patients and the active engagement of the stakeholders enhance the generalizability and relevance of the research findings. The study will produce real-world evidence on the long-term benefits and risks of these most commonly used bariatric procedures in current clinical practice.

## Abbreviations

AGB: adjustable gastric banding
BMI: body mass index
BP: blood pressure
CDM: common data model
CDRN: Clinical Data Research Network
CPT: current procedure terminology
DVT: deep vein thrombosis
EHR: electronic health record
GERD: gastroesophageal reflux disease
HCPCS: Healthcare Common Procedure Coding System
ICD-9-CM: International Classification of Diseases, 9th Revision, Clinical Modification
IRB: institutional review board
NAFLD: non-alcoholic fatty liver disease
PBS: PCORnet Bariatric Study
PCORI: The Patient-Centered Outcomes Research Institute
PCORnet: The National Patient-Centered Clinical Research Network
PCOS: polycystic ovarian syndrome
PE: pulmonary embolism
RYGB: Roux-en-Y gastric bypass
SG: sleeve gastrectomy
SNOMED: Systematized Nomenclature of Medicine
